# Editorial: Reviews in pediatric primary immunodeficiencies

**DOI:** 10.3389/fped.2025.1588443

**Published:** 2025-03-26

**Authors:** Sara Sebnem Kilic

**Affiliations:** Department of Pediatric Immunology and Rheumatology, Bursa Uludag University Faculty of Medicine, Bursa, Türkiye

**Keywords:** inborn errors of immunity, primary immunodeficiencies, Ikaros (IKZF1), immunoglobulin, chronic granulomatous disease

**Editorial on the Research Topic**
Reviews in pediatric primary immunodeficiencies

Inborn errors of immunity (IEI) increase susceptibility to infections, severe allergic disorders, and malignancies due to deficiencies in specific immune components. The International Union of Immunological Societies Expert (IUIS) Committee updated the IEI classification, encompassing 555 IEIs and 17 phenocopies due to mutations in 504 genes (1). Autoinflammation is one of the ten main groups of immune system disorders in the IUIS classification and remains at the forefront of new discoveries, with the boundaries between immunodeficiencies and rheumatology becoming increasingly blurred. Autoinflammatory disorders (AIDs) are primarily driven by innate immune system abnormalities rather than autoimmune mechanisms. They are characterized by recurrent inflammatory episodes due to immune system dysfunction, with diverse underlying mechanisms. The most well-known pathways leading to autoinflammation include inflammasomopathies, relopathies, and interferonopathies (2). This issue provides an overview of childhood AIDs, where fever and skin manifestations are often prominent symptoms, detailing the clinical manifestations, pathophysiology, diagnosis, and management of each AID syndrome.

Patients with IEI often exhibit increased susceptibility to infections; however, not all are prone to *Staphylococcus aureus* infections (3). In this special issue, Kurz et al. examine the complex relationship between *Staphylococcus aureus* virulence and host immune susceptibility in *S. aureus*-susceptible patients. While the significance of neutrophil numbers and function is well established, the role of specific cytokines, such as functional interleukin (IL)-6 signaling, is less widely recognized. This review explores host-pathogen interactions in *S. aureus* infections in susceptible individuals, potentially paving the way for more effective management and preventive treatment strategies.

In patients with severe and recurrent infections, a minimal diagnostic workup for IEI includes a complete blood count and serum immunoglobulin levels. While antibody responses to protein antigens are commonly assessed through vaccine antibodies, anti-polysaccharide IgG antibodies are not routinely measured. This limitation can lead to significant delays in diagnosing monogenic IEI, which may initially present with an impaired IgG response to polysaccharide antigens, with or without IgG subclass deficiency (4). This issue highlights the importance of evaluating IgG responses to polysaccharide antigens to prevent delays in diagnosing immunodeficiency.

Chronic granulomatous disease (CGD) primarily results from inherited defects in components of the nicotinamide adenine dinucleotide phosphate oxidase enzyme complex (5). The authors report the first case of CGD with a homozygous loss-of-function variant in the Cytochrome B-245 chaperone 1 gene (CYBC1) reported from Nepal, and draw attention to the bacterial and fungal infections that may be caused by this genetic disorder, as well as inflammatory manifestations (such as inflammatory bowel disease, acute pancreatitis, hemophagocytic lymphohistiocytosis, systemic granulomatosis).

The Ikaros family of transcription factors has important functions in immune regulation, lymphomagenesis, and the hypothalamic-pituitar*y* axis. Ikaros family zinc finger 1 (IKZF1) is a family of hemopoietic-specific zinc finger proteins that play an essential regulatory role in multiple stages of B lymphocyte development (6). IKZF1 mutations lead to CVID, a severe B cell deficiency (7). In this issue, increasing evidence of pathophysiological genotype-phenotype correlations caused by this disorder due to 6 cases with IKZF1 defects helps us understand IKAROS-associated diseases.

Immunoglobulin (IG) replacement therapy is the standard of care for immunodeficiencies that result in impaired antibody production. Intravenous (IVIG) and subcutaneous (SCIG) administration routes are commonly used, with comparable efficacy (8). Highly purified human Ig preparations are administered to restore serum IgG levels to physiological concentrations, providing broad-spectrum polyclonal antibodies for immune support. Facilitated subcutaneous immunoglobulin (fSCIG) infusion, enhanced with recombinant human hyaluronidase (rHuPH20), is an innovative treatment approach that delivers large volumes with minimal needle insertions (9). Conventional SCIG (cSCIG) therapy typically involves weekly subcutaneous injections to prevent fluctuations in IgG threshold levels (10). Furthermore, highly concentrated IgG formulations (20%) enable the administration of the required dosage in smaller volumes than less concentrated products. These formulations can be infused quickly and in larger quantities at a single site, offering a more efficient and convenient alternative to traditional subcutaneous preparations (10). While many studies have compared the safety and efficacy of various Ig administration routes, there remains a need for more research on the incidence of infections in patients receiving IG replacement therapy via intravenous, subcutaneous, and facilitated subcutaneous routes.
